# Chinese Sexual Minority Students Experiencing Microaggressions: Implications for Sexuality Education

**DOI:** 10.3390/children9091331

**Published:** 2022-09-01

**Authors:** Diana K. Kwok, Kim Kwok

**Affiliations:** 1 Department of Special Education and Counselling, The Education University of Hong Kong, Hong Kong; 2 Department of Social and Behavioral Sciences, City University of Hong Kong, Hong Kong

**Keywords:** sexual prejudice, microaggressions, Chinese LGBQ students, rights-based sexuality education, sexual minority students

## Abstract

The sexual prejudice faced by sexual minorities or lesbian, gay, bisexual, and questioning/queer (LGBQ) students has been studied extensively around the world in the last two decades; however, it has only recently received attention from Hong Kong Chinese society, specifically in relation to this subtle form of prejudice. In the last decade, there has been an increase in the amount of literature examining the experiences of individuals encountering sexual orientation microaggressions, which are defined as discrimination or sexual prejudices manifested in subtle forms, particularly when directed toward socially marginalized groups, such as sexual minority students. The current study used a qualitative descriptive approach and semi-structured interviews to explore the themes of sexual orientation microaggressions experienced by Chinese sexual minority students. Several contextual themes to categorize microaggressions emerged: (1) the approval of heteronormative culture; (2) the use of heterosexist languages; (3) the assumption of sexual abnormality; and (4) the allowing of institutionally endorsed microaggressions. The results suggest that sexual minority students in Hong Kong experience diverse forms of microaggression in schools. The implications for the need to support LGBQ students are discussed, especially in addressing sexuality education in schools and the training of school professionals.

## 1. Introduction

The inclusion of students from sexual minority communities, such as Tongzhi or lesbian, gay, bisexual, and questioning/queer (LGBQ) students (we use the acronym ‘LGBQ’ here for convenience to have the focus on sexual orientation as all informants self-identified as cisgender) in sexuality education is considered a very challenging task when it comes to the issue of achieving equal opportunities in Hong Kong Chinese society [1,2]. LGBQ students face challenges regarding being accepted in Hong Kong schools, with homophobia, heterosexism, and sexual prejudice against them not only prevailing but also being manifested overtly in the education curriculum, counseling system, school rules, and regulations [3]. However, subtle forms of sexual prejudice have not been considered. Microaggressions are defined as discrimination or sexual prejudices that are manifested in more subtle forms, particularly in the case of sexual minorities. While the Equal Opportunities Ordinances in Hong Kong currently provide protection for other minority-group students, such as members of racial minorities and those students with disabilities, LGBQ students in Hong Kong schools can be subjected to discrimination as a result of the absence of anti-discrimination laws based on sexual orientation, gender, and intersex status. A previous large-scale survey initiated by the Hong Kong government revealed that secondary schools are perceived as the most discriminatory environment for sexual minorities [4]. Previous publications have revealed that in this social context, schools are not considered harmless and safe for LGBQ and Tongzhi students, and support services are not available for them when they encounter sexual orientation harassment [3,5,6]. Furthermore, there is growing evidence that the experiences of LGBQ students have been excluded from sexuality education programs more generally; they are mostly designed from a heterosexual standpoint, without regard to their human rights [2]. Studies around the world have shown that one major concern as to why LGBQ students have been denied sexual health information is due to sexual prejudice, which manifests in the form of negative attitudes and hostility directed against sexual minorities [7]. This denial can create mental and physical health risks for such students. On the other hand, LGBQ-inclusive sexuality education may facilitate the students’ understanding of their sexual development and may thereby minimize risks [8]. The terms used to conceptualize harassment or discriminatory attitudes against sexual minorities are homophobia, heterosexism, or sexual prejudice [9]. Researchers have largely examined LGBQ students’ experiences with overt forms of discrimination over the past two decades. Nevertheless, subtle forms of prejudice and discrimination have been given less attention. These types of discriminatory attitudes, acts, and practices are defined as microaggressions [10,11]. This study utilized the concepts of sexual prejudice and sexual orientation microaggression to understand the types of subtle discrimination confronting Chinese sexual minority/Tongzhi students. The findings from this study will inform sexuality education for educators and students, specifically related to the inclusion of sexual minorities and Tongzhi students in Hong Kong.

## 2. Literature Review

### 2.1. Sexual Prejudice and Microaggressions

Herek coined the term “sexual prejudice” to describe discrimination toward Tongzhi and sexual minorities. It refers to “all negative attitudes based on sexual orientation” [9] (p. 19). Specifically, it denotes “negative attitudes toward (a) homosexual behavior, (b) people with homosexual or bisexual orientations, and (c) communities of gay, lesbian, and bisexual people” [9] (pp. 19–20). Harassment, violence, and bullying based on sexual orientation can be conceptualized as discriminatory acts or prejudicial attitudes toward an actual or perceived LGBQ or Tongzhi identity. This hostility can be expressed symbolically, physically, or verbally, with the intention of creating negative feelings, such as hatred, toward LGBQ persons [12,13]. Historically, studies have focused mainly on the overt forms of sexual prejudice, with less attention being paid to covert or subtle forms of sexual orientation-based discrimination or prejudice [10]. According to Nadal [14], microaggressions are “brief and commonplace daily verbal, behavioral, or environmental indignities, whether intentional or unintentional, that communicate hostile, derogatory, or negative slights and insults toward members of oppressed groups” (p. 23). Most likely, these are “subtle forms of discrimination, often unconscious or unintentional, that communicate hostile or derogatory messages, particularly to and about members of historically marginalized social groups.” [11] (p. 488).

### 2.2. Manifestation of Microaggressions

There has been a growing amount of literature investigating the experiences of individuals encountering sexual orientation microaggressions, which are defined as sexual prejudices in subtle forms, different from the traditional types of sexual prejudice. The traditional forms of sexual prejudice manifest at cultural and individual levels. These types of discrimination are straightforward to distinguish; for example, the use of hate speech, direct ways of sexual bullying, or organized, angry campaigns at LGBQ events [14,15,16]. Overt or explicit forms of discrimination are largely perceived to be simpler for sexual minorities to handle as the messages delivered are predictable and less confused, and sexual minorities are then able to initiate diverse strategies to cope with the predictable response or to seek rectification [16]. Covert forms of sexual prejudice and microaggressions, on the other hand, are often rendered by individuals with good intentions but who deliver prejudice and biases without awareness. Therefore, micro-aggressive messages are hidden; those who suffer from microaggression are always left with negative emotions such as invalidation or minimization or are forced into coming-out situations. Long-term suffering from microaggressions may induce mental health difficulties or inflict psychological wounds [17,18,19,20].

### 2.3. Types of Microaggressions

Sue and colleagues have listed three types of microaggressions [19,21]. The first type is microassaults, which comprise deliberate and planned forms of prejudiced attitudes or practices with the intention of subjugating the members of a marginalized group. The second type is microinsults, which are loaded with patronizing, rude, and insensitive messages, usually beyond one’s conscious awareness, that demean a person’s sexual identity. The third type is microinvalidations, which are unconscious or unintended messages that exclude or nullify the experiences of members of the oppressed group. Nadal, Issa, and colleagues listed a fourth type of microaggression in addition to the above three types [10], in the form of institutional and environmental microaggressions, expressed through the environment, institutions, policies, programs, curricula, or legislation. Shelton and Delgado-Romero suggested that microinvalidations are the most dangerous type of microaggression because they deny the reality of other groups and impose an alternate and oppressive reality on marginalized groups [22].

We found that empirical studies of microaggression have focused mostly on the North American context, despite increasing scholarship regarding sexual orientation microaggressions in the last decade. Almost all these studies have been carried out in the United States [15,22,23,24,25,26]. The results of these studies revealed that LGBQ individuals experienced incidents of subtle discrimination and microaggressions, such as a lack of recognition of sexual identity, subtle expressions of denigration with mixed messages, or stereotyping them with biased information. LGBQ students have reported being unable to obtain support from legal and school administrations. None of the above publications has addressed the Asian context, particularly the Chinese cultural context. This article will focus on the data derived from the Hong Kong Chinese context.

### 2.4. Chinese Schools and Context Barriers Faced by Tongzhi Students in Hong Kong

Based on the traditions of Confucius, a famous Chinese philosopher in Ancient Chinese history, the notion of “education for all students” is valued highly in Chinese teaching. To put this idea into modern words, this is about justice and equality for all students, with equal educational opportunities regardless of sex, gender, or sexual orientation. Regrettably, this type of justice and equality is not commonly achieved in East Asian schools, including Hong Kong. The reality is far from this ideal, particularly for LGBQ or Tongzhi students [2]. A qualitative inquiry identified LGBQ bullying, based on psychological and cultural heterosexism, in schools where teachers and adults seldom intervened in homophobic incidents and where information on LGBQ issues in school counseling programs and practices was either difficult to find or was omitted completely [27]. One study in Hong Kong on educators’ perspectives of sexual prejudice against sexual minority students found that teacher participants were uncomfortable and ambivalent when asked to express their feelings about sexual and gender minority students. Specifically, many teacher participants expressed incorrect concepts of bisexuality, intersex, and transgender identities. Second, a considerable number of participants from the education sector encountered value conflicts and were reluctant to support sexual minorities due to cultural and religious barriers. They hesitated to consider the students’ best interests when facing opposition from parents and school administrators, particularly opposition from school and parent groups with Christian affiliations [1,2]. Besides educators, other support professionals, such as social workers, also described facing difficulties when openly supporting sexual-minority clients, partly due to the religious affiliations of the schools or opposition from parents [6,28].

Chinese educators’ and school-based support professionals’ attitudes to sexual minority students have long been under the influence of both Confucian and Western religious values [3,29]. There are two main reasons for this influence. First, in many Chinese families, same-sex affection is unacceptable as it disrupts the strong Chinese Confucian family value of filial piety (Xiao). The principle of Xiao stresses that Chinese children are responsible for continuing the family line through procreation to show respect to their parents and ancestors [30,31]. Second, along with British colonial rule, which was in place for over one hundred years before 1997, came the ingrained cultural values and Christian teachings that had been practiced in Britain for centuries. Nowadays, Christianity in Hong Kong has a considerable role in influencing school programs and education policy [29]. For example, due to Hong Kong’s colonial background, public-run schools are mostly Christian-associated. Teachers and administrators have the power to implement or to omit sexuality in education topics [29]. In addressing concerns about microaggressions associated with sexual orientation-based bullying or harassment in schools, administrators and educators do not necessarily enact their ethical responsibilities to support LGBQ students. There are no codes of ethics or practice to address the possible discrimination and prejudices enacted by educators, counselors, or social workers against LGBQ students.

To provide frameworks for educators, the Council for Professional Conduct in Education (CPC) was founded in 1994 to set up the Code for the Education Profession of Hong Kong [32]. It is stipulated in the CPC Code that educators and other professionals in schools: “shall not discriminate against any student on the basis of race, color, religious belief, creed, sex, family background, or any form of handicap” [32] (p. 1). However, sexual diversity has not been addressed in the Code. The same is true of the school social workers’ code of ethics. Although the Code of Practice from the Hong Kong Social Workers’ Registration Board states that social workers should respect the “dignity of every human being irrespective of one’s … sexual orientation” [33] (p. 1), there is no mandated training or policy to prevent social workers’ sexual prejudices against sexual minorities. Regarding the prevention of prejudice or discrimination against minority or marginalized groups through anti-discrimination law, the Equal Opportunities Commission (EOC), established in May 1996, is a statutory body working toward the elimination of discrimination on the grounds of sex, marital status, pregnancy, disability, family status, and race, but does not address sexual orientation, gender identity, and intersex status. The currently implemented Ordinances in Hong Kong include the Sex Discrimination Ordinance, the Disability Discrimination Ordinance, the Family Status Discrimination Ordinance, and the Race Discrimination Ordinance (RDO). Proposals for an anti-discrimination ordinance based on sexual orientation have been the subject of heated debate in Hong Kong in the last 20 years. This ordinance still cannot be enacted because of strong opposition from religious organizations and parents’ and teachers’ groups [31,34].

## 3. Purpose and Research Question

No publications on sexual orientation microaggressions have addressed Tongzhi/LGBQ students in non-Western contexts, particularly in the Hong Kong Chinese cultural context. This study aimed to fill this knowledge gap. The rationale was that it is difficult for educators or students to identify subtle or covert forms of sexual prejudice and sexual orientation microaggressions; hence, it will be helpful for school personnel to understand LGBQ microaggressions as a means to design support programs for LGBQ students. Thus informed, educators may also provide adequate training for other students and help professionals who are likely to interact with LGBQ students; these people may themselves be the perpetrators of microaggressions, therefore, preventive measures and education are needed. The research question was: What are the forms of microaggression experienced by LGBQ students in Hong Kong? The findings from this study will inform both sexuality education and educators’ training related to the inclusion of LGBQ students.

## 4. Methodology

The transcripts for this article were collected while the researchers were conducting a research project on sexual prejudice and sexuality education in Hong Kong, with the aim of exploring the experiences of minority students in schools. This paper focused on transcripts from fifteen sexual minority students. To answer the research question of this paper, the “qualitative description approach” was adopted [35] (p. 867). This qualitative approach has been applied in many health studies [35,36,37]. It is “ideal for effectively identifying observations and constructs emerging from narrative-based data” and is not aiming at “a specifically defined lens or theoretical approach to data interpretation”. In line with this approach, purposive sampling was used for sourcing in-depth information [35] (p. 867).

### 4.1. Recruitment and Participants

Ethical approval was granted by the Education University of Hong Kong’s Human Research Ethics Committee. In addition, parental consent waivers for participants aged 16 to 17 were obtained. We recruited participants from diverse networks, including youth queer groups, mutual help groups, and counseling centers that run sexual and gender diversity services. The criteria for selecting the research participants were: (1) demonstrating same-sex attraction/behaviors/experiences and/or self-identifying as LGBQ/Tongzhi/sexual minorities; (2) having encountered LGBQ-related sexual prejudice (either covert or overt)/harassments; (3) attending secondary schools or having left them within the previous five years; and (4) being comfortable enough to express their feelings and being interested in their experience. Fifteen participants were recruited. Among them, five participants self-identified as lesbians, four identified as bisexuals, and six identified as gay men. The participants ranged from 17 to 22 years of age. Six participants were attending high schools, while the remaining participants were attending college.

### 4.2. Data Collection

Interviews were conducted in private locations, ensuring discretion and confidentiality. Ethical approval was obtained before the interviews. Each interview lasted around 1.5 to 2 h. The participants were given information beforehand about the research background and process. They were encouraged to raise questions to clarify the research procedures. With the support of a semi-structured interview guide [38], the interviews were mostly collaborative and interactive. Examples of semi-structured questions included: What are sexual prejudices, especially those targeting Tongzhi/LGBQ individuals, in direct and/or indirect/subtle forms? Share with us experiences/incidents that are related to your sexual development/sexuality/sexuality education in schools; how did the school react to these incidents? Since the issue of an imbalance of power between the researchers and the participants can arise in qualitative studies, the interviewers would share their experiences of working with Tongzhi/LGBQ communities whenever necessary and would provide affirmative feedback throughout the process, which facilitated a trusting and safe atmosphere for in-depth sharing.

### 4.3. Data Analysis

Qualitative thematic analysis was used to conduct data analysis [38,39,40,41,42]. The goal was to find out the forms or patterns of microaggressions that people had heard, overheard, and experienced regarding non-heterosexual identities. The process involved the researchers lessening their personal bias through self-reflection by keeping field notes and holding peer meetings to discuss biases and hypotheses that could play a role in the data analysis process. The first stage involved the immersion of researchers and a research assistant in the transcripts, to gain a sense of familiarity with the stories told by the participants. At the same time, initial ideas of potential codes were reflected, based on field experience and meeting discussions [38,42,43]. In the second stage, the general impressions of LGBQ students’ discriminatory experiences, including both overt and subtle forms of discrimination, were identified through the researchers’ reading of the interview transcripts. In the third stage, a coding framework was set up based on the themes abstracted from the first few transcripts and meeting discussions. In the fourth stage, themes within the interview transcripts were identified and extracted according to the coding framework, using NVivo. The fifth and final stage involved the defining and final naming of themes. The coded themes were then reviewed again and again to assess whether the themes were consistently well-developed. Tactics employed to safeguard the rigor of this research were: the management of subjectivity and reflexivity throughout the research process; obtaining rich descriptions of the participants’ responses, prolonged engagement in the sexual minority communities; the use of peer debriefing, participants’ feedback, and persistent observation [38,42].

## 5. Results

The participants reported experiences centered around the following themes: (1) approving heteronormative culture and invalidating non-heterosexuality; (2) the use of heterosexist language; (3) the assumption of sexual abnormality and endorsement of stereotypes; (4) the allowing of institutionally endorsed microaggressions.

### 5.1. Approving Heteronormative Culture and Invalidating Non-Heterosexuality

This theme arises when sexual minorities are expected to be heterosexual or to act as heterosexuals. The participants discovered that non-heterosexual identities are often looked down upon or treated as being less legitimate than heterosexual identities. The experiences of repeatedly revealed forms of microaggression, as shared by the participants, included minimizing or nullifying their sexuality, invalidating their experiences of sexual development or sexual identities, and perceiving these experiences as a passing phase and untrue. One bisexual female student, Ivy, remembered the following experiences:

“In my school, which is Christian-affiliated, a poster I saw outside the classroom, saying: ‘We insist on once in a lifetime, one husband and one wife, one man and one woman’. Our sexuality education was all around the theme of preparing us to be good wives in society as if an individual’s identity ‘should’ be as a man’s ‘future wife’. From my perspective, sexuality/ies for many girls around me are fluid, and some of my friends are lesbians or even transgender women who love girls, what about those girls who fall in love with girls?”

Some participants shared that other students and teachers often responded to their questions from a heterosexual perspective, seeing their dating experiences as being less valid than those of their heterosexual peers. A lesbian participant, Jenny, told the story:

“I would not come out when I had a crush on a girl in my school. What should I do? Hide my identity? I knew that there was this girl who came out as a lesbian and was dating another girl. The teachers worked hard to break apart this relationship, to prevent them from meeting each other. How could you come out after that incident?”

One gay student recalled his experience of meeting a guidance counselor about exploring his struggle with having sexual and affectionate attraction toward boys. He said:

“I am interested in dating boys. I came to talk to my counselor. The counselor was affiliated with a Christian agency in Hong Kong. He said I cannot breach God’s wish. He repeatedly shared the perspective that being a gay man is only a developmental ‘stage’ for me, and I will become ‘straight’ in the future, such as in the university stage. Now, I am in the university, I am still attracted to men. Yet at the time of secondary school, while I was exploring myself, I heard that message … that information he provided was wrong. It was disturbing when someone with ‘professional’ authority said that.”

### 5.2. The Use of Heterosexist and Abusive Language

The Tongzhi students described having overheard or personally received comments that were obviously offensive to LGBQ individuals. These included explicit and intended verbal attacks, insults, and derogations, as manifested through discriminatory practices and name-calling toward those individuals. The participants also experienced microaggressions in the form of heterosexist language and terminologies that privileged heterosexuality. These verbal and physical insults, according to Nadal et al. [10], are microassaults with the intention to suppress sexual minority students. One gay participant shared his experience:

“Looking back to my school days, I was always teased by my peers in my junior years of secondary school as they perceived me as a non-straight ‘sissy’ boy. They went further and accused me of being gay. I was intentionally picked on with verbal assaults by some bullies because I ‘act like a girl’, as they said, and was regarded as ‘sissy’. They would not pick on classmates who looked straight or had strongly built bodies. Instead, they would turn and stalk me at bus stops or throw away my school bag, particularly when I talked back. I felt ashamed and humiliated to be condemned this way simply because of my sexual orientation.”

The participants said that they had overheard such remarks in schools and felt frustrated that these microassaults seemed to be acceptable in school. According to the participants, facing these incidents or hearing abusive comments was threatening, uncomfortable, and hurtful.

### 5.3. The Assumption of Sexual Abnormality: Endorsing Stereotypes

The participants had experienced the endorsement of stereotypes and sexual prejudice wherein some educators and students portrayed sexual minorities as morally, and/or emotionally, unnatural. Messages or comments that gay and lesbian intimate relationships and sexual identities are less legitimate, healthy, or natural than those with heterosexual partners were often overheard or received. The participants also felt that some teachers and peers thought it was their concern to give suggestions or to give warning signals about the essential threats linked with coming out or expressing sexual identity. They may feel the need to caution students about coming out. Here is an example of a gay man’s description of his “coming-out” to a teacher who uttered stereotypical comments, despite not intending to hurt the student:

“I had an experience with my favorite teacher. When I decided to come out, I first came to her, whom I expected to be there for me … to my disappointment, she said that I may not be sure what I am getting into … and this is a developmental stage (falling in love with boys), and this is not a healthy lifestyle. I might fall in love with girls in the future, at the end, she said, it’s better to fall in love with a girl. I felt very bad about this coming-out experience, as she had stereotypical concepts about sexual orientation, and without awareness that she had induced hurtful feelings in her students.”

One gay student recalled the following incident:

“One teacher talked about a Bible story, which was about God demolishing a town because there were sins, and one of the sins was homosexuality. In the sexuality education workshop, the invited guest speaker talked about sexuality education based on religious and abstinence principles, that unwed pregnancy should be avoided, and their assumptions were taken from a heterosexual framework.”

One lesbian participant shared that:

“Some peer students assumed that lesbian students, such as those who are ‘tomboys’, are cutting their hair short due to the influence of popular culture. They are merely acting out these ‘attention-seeking’ behaviors to attract adults’ attention.”

A bisexual participant felt pressured to educate others about bisexuality when her peers sometimes asked questions about her sexual and romantic relationships. She said:

“These questions offended me. I was not comfortable having to answer, and my emotional response was minimized for the sake of having other students get opportunities to learn about bisexual culture.”

### 5.4. Allowing Institutionally Endorsed Microaggressions

The participants described that microaggressions came not only from individual students, teachers, school social workers, or administrators but were also expressed through school surveys, leadership, or teaching curricula. One bisexual student expressed her experience thus:

“On the student intake form for the counseling service, on the item asking about sex, there are only the options of either male or female but no room for intersex, or transgender. No question on sexual orientation can be found on the form. The principal in my school, which is a Catholic school, thought that homosexuality does not exist. There are no guidelines from the Education Bureau on how to handle it.”

Another gay student commented:

“The sexuality education curricula focused on abstinence education, such as not having premarital sex, or how to avoid sexually transmitted diseases, etc. The female classmates and male classmates were separately engaged in two different rooms. Teachers were not allowed to talk about sex or dating outside marriage. My teacher once told me that they could not afford to face the consequences, such as pressures from the parent-school association members and the school administrators, such as the principal, or complaints from the parents.”

Some participants mentioned sexuality education workshops or curricula containing microaggressions relating to sexual orientation. For example, some religious schools invited pastors to conduct sexuality education workshops, in which heterosexuality was perceived as the only legitimate framework. One participant shared that the guest speaker invited by the school talked about sexuality education based on abstinence principles, arguing that unmarried pregnancy should be avoided. Other than that, in some instances, participants found that schools encouraged the manifestation of microaggressions by failing to set up a system that would challenge them.

## 6. Limitations

The study has limitations. It aimed at understanding the types of microaggressions encountered by a particular group of students through obtaining information-rich narratives. It did not intend to generalize their experiences in relation to other LGBQ groups. In addition, it did not address intersectional variations in socio-economic status, race, disability, age, gender, etc., in the lived experiences of sexual minority students. It is likely that these variables and the privileges/oppressions associated with them would have had an impact on the participants’ sexual explorations, identity expressions, and views of the school environment, as well as the strategies that they adopted to respond to school microaggressions. This paper reports the participants’ additional insights into how sexual orientation microaggression, a subtle form of prejudice, is manifested within the Chinese school context in Hong Kong.

## 7. Discussion

Regardless of the limitations, this study adds to the existing body of literature on sexual orientation microaggression. It found that sexual orientation microaggressions were manifested in various forms, namely, microassaults, microinsults, microinvalidations, and institutional microaggressions. The findings are comparable with those articulated in previous studies [10,22]. Some teaching professionals, such as administrators and educators, not only neglect the sexual development of Tongzhi students but also reinforce microaggressions and heterosexism when interacting with Tongzhi students or when implementing sexuality education curricula and school practices [10,44]. The findings also show that LGBQ-related microaggression in school can engender additional minority stress on students and their emotional well-being [17,18,19,20,21,45]. While corresponding findings have been reported about the various forms of microaggressions in the United States, the situation appears to be tougher for Tongzhi youth in societies such as Hong Kong, where schools have not yet established support programs for LGBQ students to meet the challenges of sexual prejudice, in addition to the lack of legal support. We found a study on sexual prejudice in Latin American cultures, where people are “generally more attached to traditional values emphasizing the importance of religion and family” [46] (p. 1550), with similar cultural themes to those we observed in Hong Kong. The research results from Villar and colleagues conducted in Latin American countries suggested that attitudes to sexual minorities are “more favorable in those countries in which efforts have been made in favor of equality [and the] public recognition of rights” [46] (p. 1565). In Hong Kong, the recognition of Tongzhi rights through an anti-discrimination ordinance is not available. Other studies have found that those students enrolled in schools that have school-based supportive programs, like the Gay-Straight Alliance, experience mental health benefits [47,48,49]. However, the wider socio-cultural climate of Hong Kong is largely heteronormative, including in the education system. Schools have many barriers to establishing these school-based programs [2,6]. Up until the date of the study, Christian teaching has still had a strong influence on the education system and government policies. Several Christian groups oppose the proposed enactment of the Anti-Discrimination Ordinance on sexual orientation, arguing that homosexuality is sinful. At the same time, some parents’ groups are also allied with Christian groups to oppose the giving of rights of LGBQ individuals [34]. Therefore, cultural, and religious impacts may create barriers for schools, teachers, and school social workers/counselors when trying to support sexual minority students. Without anti-discrimination laws and explicit codes of ethics to prevent sexual prejudice, educators, social workers, and counselors in schools are facing institutional barriers to running support programs for Tongzhi students. In contrast to disability and racial minority students, who are protected legally under the Anti-Discrimination Ordinance and other inclusive educational policies, the principle of equality and inclusive educational policies has not been applied to sexual minority students to protect them from LGBQ-related harassment and discrimination.

## 8. Recommendations

Troutman and Packer-Williams observed that the failure to include the concerns of the LGBQ population in training/education may be considered “a form of systemic prejudice or discrimination” [49] (p. 7). Sexuality educators are expected to play crucial roles in supporting LGBQ students to navigate sexual prejudice, microaggressions, and mental health risks, through LGBQ-inclusive and prejudice-free sexuality education [50,51]. One emerging framework for sexuality education is the rights-based model, which integrates human rights principles into sexuality education [52,53]. For example, the Yogyakarta Principles identify human rights principles and their application to LGBQ issues for sexuality education [51,54]. Principle 3 indicates the “right to recognition” before the law, in accordance with self-defined gender identity [54] (p. 11). The recently added Principle 16 highlights the addition of “affirmative and accurate material” on sexuality education for LGBQ children and youth. Rights-based sexuality education is, therefore, crucial to acknowledging the human rights of LGBQ students to access relevant sexuality education. Yet, in Hong Kong, the government’s progress in enacting legal reforms to protect sexual and gender minorities’ human rights has been “seized upon by counter-movements, including religious opposition and parental concern groups” [34] (p. 138). To work within these cultural and religious barriers, a few recommendations are suggested. First, the Government departments, including the Education Bureau (for initiating educators and school counselors’ training), and the Social Welfare Department (for initiating school social workers’ training) should take the lead in initiating sexuality education for educators and school social workers, creating a training curriculum involving anti-prejudice sexual diversity content. School administrators are in a better position to support their teachers and school social workers to enroll in the training workshops that are offered by training institutes, or to start such training plans in schools within the teachers’ professional development context [1]. Additionally, Tongzhi/LGBQ individuals, including students from the education setting, do not have a similar level of protection under the Equal Opportunities Ordinance as their heterosexual peers. They may face marginalization, isolation, and mental health risks [3,30] within the existing heterosexist-based education context. LGBQ-inclusive sexuality education could be incorporated into the general education and civic education curriculum within the curriculum guidelines of the Education Bureau. In support of equal opportunities for all students, the schools’ social workers and counseling teams may consider commencing actions to educate students about respecting sexual diversity through projects showing zero tolerance for homophobic harassment. Finally, in terms of future research, comprehensive, quantitative research is recommended to increase our understanding of microaggressions in education settings. In addition, studies investigating issues with topics such as teachers and social workers acting as allies will offer valuable data to design support and education practices from the point of view of empowerment and social justice.

## 9. Conclusions

Regardless of the research limitations, the present research study adds to the existing body of work on microaggressions encountered by Tongzhi students in the Hong Kong Chinese cultural context. Without the existence of the Anti-Discrimination Ordinance based on Sexual Orientation, sexual minority and Tongzhi students face homophobic microaggressions without receiving institutional support from equal-opportunity school policies or professional support from educators and social workers. This also reveals an urgent need for educators and professionals to support Tongzhi students facing sexual prejudice along with unique cultural barriers, manifested through different forms of discrimination, to enhance their professional competence in addressing cultural barriers and offer better support to Tongzhi students. Educators and support professionals are in a good position to challenge sexual prejudice, and society’s stereotypes, and biased assumptions regarding sex, gender, and sexual orientation. Social workers, counsellors, and educators as support professions, will fail in their missions if respect for and tolerance of sexual diversity are not advocated.

## Data Availability

The data are not publicly available due to ethical restrictions.
